# The effect of breathing on ductus arteriosus blood flow directly after birth

**DOI:** 10.1007/s00431-017-2994-9

**Published:** 2017-09-07

**Authors:** Jeroen J. van Vonderen, Arno A.W. Roest, Frans J.C. Klumper, Stuart B. Hooper, Arjan B. te Pas

**Affiliations:** 1 Division of Neonatology, Department of Pediatrics, Leiden University Medical Center, J6-S, PO Box 9600, 2300 RC Leiden, the Netherlands; 2 Division of Pediatric Cardiology, Department of Pediatrics, Leiden University Medical Center, Leiden, the Netherlands; 3 Department of Gynecology and Obstetrics, Leiden University Medical Center, Leiden, the Netherlands; 4 The Ritchie Centre, MIMR-PHI, Monash University, Clayton, Victoria Australia

**Keywords:** Neonatal hemodynamics, Crying, Ductus arteriosus, Breathing, Pulmonary blood flow

## Abstract

Spontaneous breathing at birth influences ductus arteriosus (DA) flow. This study quantifies the effect of breathing on DA shunting directly after birth. In healthy term infants born by elective cesarean section, simultaneous measurements of DA shunting and tidal volumes during spontaneous breathing were performed at 2–5, 5–8, and 10–13 min after birth. Eight infants with a mean (SD) gestational age of 40 (1) weeks and 3216 (616) grams were studied. Inspiratory tidal volume was 5.8 (3.3–7.7), 5.7 (4.0–7.1), and 5.2 (4.3–6.1) mL/kg at 2–5, 5–8, and 10–13 min. The velocity time integral of left-to-right shunting significantly increased during inspiration when compared to expiration (8.4 (5.2) vs. 3.7 (2.3) cm, 8.9 (4.4) vs. 5.6 (3.4) cm, and 14.0 (6.7) vs. 8.4 (6.9) cm; all *p* < 0.0001) at 2–5, 5–8, and 10–13 min, respectively. In contrast, right-to-left shunting was not different between inspiration and expiration at 2–5 and 10–13 min (11.1 (2.4) vs. 11.1 (2.6) cm and 10.7 (2.3) vs. 10.6 (3.0) cm; *p* > 0.05), but there was a small increase at 5–8 min (12.1 (2.4) vs. 10.8 (2.9) cm; *p* = 0.001) during expiration.

*Conclusion*: Directly after birth, ductal shunting is influenced by breathing effort, predominantly with an increase in left-to-right shunt due to inspiration.

**Table:** 

**What is Known:** • Spontaneous breathing at birth influences ductus arteriosus flow and pulmonary blood flow. • Crying causes a significant increase in left-to-right ductus arteriosus shunting.	
**What is New:** • Left-to-right ductus arteriosus shunting increases during inspiration compared to expiration. • Breathing is important for ductal shunting and contributes to pulmonary blood flow.

## Introduction

Directly after birth, lung aeration has a large impact on the changes that take place in both the hemodynamic and respiratory systems [6, 9]. Lung aeration causes a decrease in pulmonary vascular resistance (PVR) and an increase of pulmonary blood flow (PBF), of which in lambs 30–50% originates from left-to-right (LtoR) shunting through the ductus arteriosus (DA) [1]. We recently observed that in infants, in the first 10 min after birth, DA shunting changes from predominantly right-to-left (RtoL) to predominantly LtoR [10]. The increase in DA LtoR shunting increases preload contributing to a 25% increase in left ventricular output shortly after birth [9]. We also observed that the large sub atmospheric pressures during the inspiratory phase of crying significantly increased LtoR shunting, most probably because it causes large decreases in transpulmonary pressure [11].

In the fetus, large inspiratory efforts are known to decrease PVR and increase PBF [4] and so the large inspirations that commonly precede crying could transiently decrease PVR and change the direction of DA blood flow from predominantly RtoL to predominantly LtoR [11]. However, the effect of breathing on DA blood flow directly after birth has not been quantified. We aimed to quantify the change in DA shunt flow and its relationship with the inspiratory and expiratory phases of the respiratory cycle during spontaneous breathing in term infants directly after birth.

## Methods

Healthy term infants (≥ 37 weeks of gestation) delivered by elective cesarean section between February 2014 and September 2014 were included in this study. The study was approved by the Institutional Review Board of the Leiden University Medical Center. Parental consent was obtained during pre-operation visit or by telephone at least 1 day before the cesarean section was performed.

Only uncompromised infants born after elective cesarean sections, without congenital malformations, were included in this study, as these infants were routinely evaluated on a resuscitation table. Infants born by vaginal delivery were directly placed on the chest of the mother and so were not available for study. The non-invasive measurements performed did not influence standard care. Infants were excluded when transition at birth was complicated, and respiratory support was needed.

A stopwatch was started as soon as the infant was born (i.e., when the shoulders and head were delivered). In accordance with local guidelines, the umbilical cord was clamped 30–60 s after birth and cut by the obstetrician. Therefore, most infants commenced breathing before clamping of the umbilical cord. Thereafter, the baby was placed under a radiant heater and the neonatal caregiver, who was not involved in the research project, provided standard care (drying, keeping warm, evaluation of the infant’s condition). Stimulation, positioning, and suctioning were left at the discretion of the neonatal caregiver. Measurements were collected at three time points after birth as soon as the infant was placed on the resuscitation table and at 5 and 10 min after birth. During this period, the infant remained on the resuscitation table.

Electrocardiography (ECG) electrodes (Neotrode II; Conmed, Uticofa, N.Y., USA) were placed as soon as the infant reached the resuscitation table to determine heart rate (HR), and the ECG was recorded simultaneously with the echocardiographic examination.

To measure tidal volumes a face mask (Laerdal 0/1 round face mask, Laerdal, Stavanger, Norway) and an attached flow probe with a hot wire anemometer (Florian, Acutronics AG, Hirzl, Switzerland) were used. The face mask, open to air, was gently placed on the infants face using the two-point top hold [12], which has previously been shown to have little influence on breathing [7]. The gas flow signal was recorded at 200Hz and integrated to provide inspired (Vti) and expired tidal volumes (Vte) using Spectra software (Spectra, Grove Medical Limited, Hampton, UK). To measure the effect of the respiratory effort, we also determined the rate of rise to maximum tidal volume (mL/kg/s) for inspiration and rate of decrease to minimal tidal volume (mL/kg/s) for expiratory tidal volume.

To assess DA shunting, an echocardiographic examination was performed using a Vivid 7 Cardiovascular Ultrasound system equipped with a 7.0-MHz transducer (GE Healthcare, Waukesha, Wisc., USA). The DA velocity time integral (VTI) was assessed using 2D echocardiography and Doppler continuous wave measurements at the DA in the suprasternal view. Doppler images were collected in synchrony with respiratory function monitoring and visualized in the spectra program to insure synchrony of data analysis. VTIs of LtoR and RtoL shunting and Vmax of both shunt directions were calculated.

To prevent inter-individual variation between observers, all echocardiograms and measurements were performed by one senior pediatric cardiology consultant. All gathered data was analyzed by one researcher. All infants received a comprehensive echocardiogram after the procedure to rule out any structural anomalies of the heart.

### Statistical analysis

Data were analyzed using SPSS (version 20.0.0; IBM, Chicago, Ill., USA). Results are presented as mean (SD), mean (range), or median (IQR) as appropriate. Paired normal distributed continuous data was compared using a paired *t* test. Paired non-normal distributed continuous data was compared using a Wilcoxon signed rank test. A *p* value < 0.05 was regarded as statistically significant. Reported *p* values are two-sided.

## Results

Measurements were obtained from nine infants, although one infant needed respiratory support and was excluded from analysis. The eight included infants had a mean (SD) gestational age of 40 (1) weeks and 3216 (616) grams and a median (IQR) Apgar score of 9 (9–9) at 1 min, 9 (9–10) at 5 min, and 9 (9–10) at 10 min after birth. A total of 536 breaths with concomitant ductal flow patterns were analyzed.

**Fig. 1:**
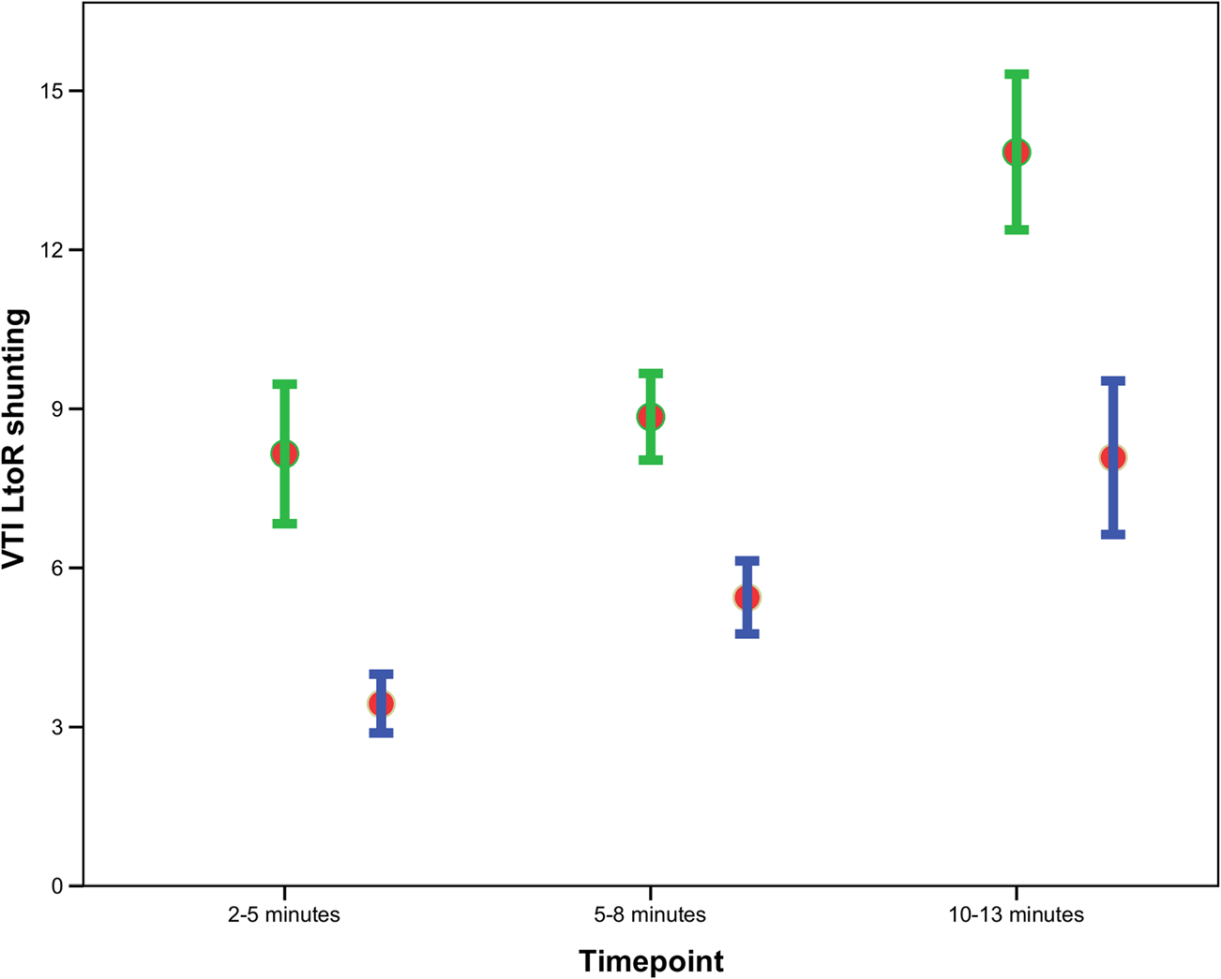
VTI of left-to-right shunting during inspiration (green bars) and expiration (blue bars) at 2–5, 5–8, and 10–13 min after birth

Inspiratory tidal volume was 5.8 (3.3–7.7), 5.7 (4.0–7.1), and 5.2 (4.3–6.1) mL/kg at 2–5, 5–8, and 10–13 min, respectively. Expiratory tidal volume was 5.1 (2.7–7.0), 4.9 (2.0–7.3), and 5.2 (4.3–6.1) mL/kg at 2–5, 5–8, and 10–13 min (Fig. 1). Rate of rise to maximal tidal volume increased over time from 12.6 (6.8–22.9) to 14.3 (8.9–22.6) to 15.9 (11.0–23.9) mL/kg/s (*p* = 0.05) at 2–5, 5–8, and 10–13 min, respectively. Rate of decrease to maximum expiratory tidal volume increased significantly from 5.5 (3.0–15.4) mL/kg/s at 2–5 min to 13.4 (4.3–22.1) mL/kg/s at 5–8 min (*p* = 0.01) and then decreased to 8.4 (6.0–14.6) mL/kg/s at 10–13 min (*p* > 0.05).

At all-time points, a bidirectional flow pattern over the DA was observed with right-to-left shunting during systole and left-to-right shunting during diastole. VTI of LtoR shunting was significantly larger during inspiration when compared to expiration (8.4 (5.2) vs. 3.7 (2.3), 8.9 (4.4) vs. 5.6 (3.4), and 14.0 (6.7) vs. 8.4 (6.9) cm; *p* < 0.0001) at 2–5, 5–8, and 10–13 min, respectively. In contrast, VTI of RtoL shunting was not significantly different between inspiration and expiration at 2–5 and 10–13 min (11.1 (2.4) vs. 11.1 (2.6) cm and 10.7 (2.3) vs. 10.6 (3.0) cm; *p* > 0.05). However, at 5–8 min, VTI of RtoL shunting was significantly higher during expiration (12.1 (2.4) cm) as compared to inspiration (10.8 (2.9) cm; *p* = 0.001).

Vmax of LtoR shunting was significantly larger during inspiration when compared to expiration (0.69 (0.22) vs. 0.43 (0.18) cm/s, 0.65 (0.24) vs. 0.46 (0.21) cm/s, 0.76 (0.23) vs. 0.52 (0.24) cm/s; (*p* < 0.0001) at 2–5, 5–8, and 10–13 min, respectively. Vmax of RtoL shunting was not significantly different between inspiration and expiration at 2–5 and 10–13 min (1.00 (0.19) vs. 0.97 (0.18) cm/s and 0.99 (0.12) vs. 0.95 (0.16) cm/s; (*p* > 0.05), but at 5–8 min, Vmax of RtoL shunting was significantly higher during expiration (1.03 (0.16) cm/s) compared to inspiration (0.95 (0.18) cm/s; *p* = 0.001).

## Discussion

We observed that, in breathing infants directly after birth, LtoR shunting through the DA was significantly increased during inspiration as compared to expiration, while no changes were observed in RtoL shunting. Despite the finding that tidal volumes decreased over time, we found that the VTI of LtoR shunting and the difference between inspiration and expiration increased over time. These findings confirm that breathing has a predominant effect on LtoR ductal shunting and contributes to an increase in PBF. This implies that the sub atmospheric pressure produced during spontaneous inspiration facilitates the hemodynamic transition at birth [11].

This is the first study showing the direct effect of breathing on DA flow during quiet breathing in newborn infants. We have previously described the effects of large inspiratory efforts during crying, which causes large sub atmospheric intra thoracic pressures and transient reductions in PVR to promote LtoR shunting and increase PBF [11]. While a transient reduction in PVR may be the predominant driver for the increase in LtoR shunting, the reduction in intra thoracic pressure will also greatly reduce the pressure gradient for blood to flow from intra thoracic and into more distal extra thoracic arteries. As such, this will encourage output from the left ventricle to flow LtoR through the DA rather than into extra thoracic arteries. The influence of breathing on DA flow has been described in various experimental studies [2, 3, 8]. Iwamoto has shown that LtoR shunting is decreased in fetal lambs during positive pressure ventilation compared with spontaneous breathing [3] which reflects the well-described effects of applying supra atmospheric pressures on increasing PVR and systemic venous return. Indeed, excessively high PEEP levels are known to reinstate RtoL shunting in newborn lambs [5].

In a group of healthy term infants born after a cesarean section, we have shown that left ventricular output increased by 25% between 2 and 5 min after birth [9] due to an increase in stroke volume resulting from an increase in pulmonary venous return and subsequently left ventricular preload. The increase in PBF and pulmonary venous return is in part derived from the LtoR DA shunting which in lambs increases by 50% between 2 and 10 min after birth (3) and is also increased further during inspiration. As intra thoracic pressures strongly influence LtoR shunting, PBF, pulmonary venous return, and left ventricular output, clearly positive pressure ventilation has a significantly different effect on the cardiovascular transition compared with spontaneous breathing at birth. Furthermore, the temporarily promotion of LtoR blood flow through the DA directly after birth could lead to a reduction of umbilical arterial flow after birth and eventually contributes the cessation of flow.

The increase in LtoR shunting while RtoL shunting remains constant is in agreement with our previous work [10]. As the lungs become more aerated, PVR decreases and as a consequence, the pressure gradient between the pulmonary circulation and systemic circulation reverses causing a predominant LtoR shunt [10]. The increase in LtoR DA shunting during inspiration is partly caused by a higher Vmax possibly explained by the increased rate of rise at all-time points. During RtoL DA shunting, Vmax was only significantly different between inspiration and expiration at 5–8 min after birth causing a significantly different RtoL VTI. Although the differences between inspiration and expiration were not significantly different between 2 and 5 and 10–13 min, we speculate that the increase in RtoL shunting could be caused by a more forceful expiration as explained by the high rate of rise at this time point. As a result, there will be an increase of intra thoracic pressure and RtoL DA shunting.

DA shunting is influenced by various parameters which include (1) pressure changes, for example, intra thoracic pressure changes during both inspiration and expiration which in turn is determined by both inspiratory and expiratory effort and (2) hemodynamics, which include pulmonary venous return, heart rate, cardiac output, and peripheral vascular resistance. This causes a pressure gradient over the DA [9]. We speculate that the influence of these other parameters will have more influence on DA shunting after the immediate changes that take place in the first 10–15 min after birth. In the first 10 min after birth, the influence of breathing and its effect on intra thoracic pressure is probably the most important for DA hemodynamics due to the large diameter of the DA which thereafter quickly decreases in healthy term infants [10]. Unfortunately, we were not able to make recordings later than 13 min after birth to study this effect. The small sample size and the non-blinded collection of data and its analysis are the limitations of this study. Unluckily, we were not able to include more patients due to the introduction of the family centered cesarean section during which the infant remains with the mother. However, the effect of breathing on DA blood flow shunting was found to be highly significant and variation was small. In this study, we only included infants born by cesarean section who could have had a delayed transition. However, all infants breathed spontaneously with tidal volumes that were considered adequate.

## Conclusion

We observed that during quiet breathing inspiration causes a significant increase in LtoR DA shunt compared to expiration during the first minutes after birth. The increase in DA shunting is probably caused by larger sub atmospheric pressures as there is a significant correlation with the respiratory effort. RtoL DA shunting is only marginally influenced by breathing. The increase in LtoR shunting could be favorable for hemodynamic transition, and therefore, spontaneous breathing should be stimulated during neonatal transition.

## Abbreviations

DA: ductus arteriosus
ECG: electrocardiography
LtoR: left-to-right
PBF: pulmonary blood flow
PVR: pulmonary vascular resistance
RtoL: right-to-left
VTI: velocity time integral
